# Antifungal activity of compounds from *Gordonia* sp. WA8-44 isolated from the gut of *Periplaneta americana* and molecular docking studies

**DOI:** 10.1016/j.heliyon.2023.e17777

**Published:** 2023-07-04

**Authors:** Wenbin Liu, Ertong Li, Lingyan Liu, Fangyuan Tian, Xiongming Luo, Yanqu Cai, Jie Wang, Xiaobao Jin

**Affiliations:** a Guangdong Provincial Key Laboratory of Pharmaceutical Bioactive Substances, Guangdong Pharmaceutical University, Guangzhou 510006, PR China; b School of Pharmacy, Xi'an Medical College, Xi'an 710300, PR China; c School of Basic Medical Sciences, Guangdong Pharmaceutical University, Guangzhou 510006, PR China

**Keywords:** Antifungal, *Periplaneta americana*, *Gordonia*, Molecular docking, Collismycin A

## Abstract

Invasive fungal infections are on the rise, leading to a continuous demand for antifungal antibiotics. Rare actinomycetes have been shown to contain a variety of interesting compounds worth exploring. In this study, 15 strains of rare actinobacterium *Gordonia* were isolated from the gut of *Periplaneta americana* and screened for their anti-fungal activity against four human pathogenic fungi. Strain WA8-44 was found to exhibit significant anti-fungal activity and was selected for bioactive compound production, separation, purification, and characterization. Three anti-fungal compounds, Collismycin A, Actinomycin D, and Actinomycin X_2_, were isolated from the fermentation broth of *Gordonia* strain WA8-44. Of these, Collismycin A was isolated and purified from the secondary metabolites of *Gordonia* for the first time, and its anti-filamentous fungi activity was firstly identified in this study. Molecular docking was carried out to determine their hypothetical binding affinities against nine target proteins of *Candida albicans*. Chitin Synthase 2 was found to be the most preferred antimicrobial protein target for Collismycin A, while 1,3-Beta-Glucanase was the most preferred anti-fungal protein target for Actinomycin D and Actinomycin X_2_. ADMET prediction revealed that Collismycin A has favorable oral bioavailability and little toxicity, making it a potential candidate for development as an orally active medication.

## Graphical abstract

**Figure:**
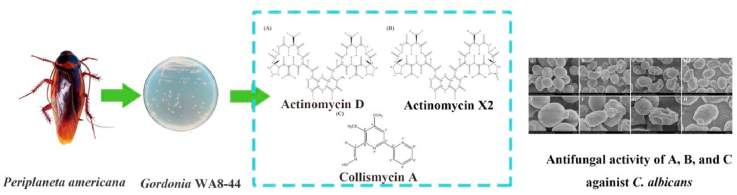

## 1 Introduction

The incidence of invasive fungal infections is on the rise due to an increase in the number of immunocompromised patients [1] and the emergence of antibiotic-resistant strains, particularly those resistant to azole drugs [2]. There is an urgent need to develop new anti-fungal antibiotics with novel mechanisms of action to efficiently combat these infections [3]. To address this challenge, researchers have undertaken a diverse range of approaches, including the discovery of new bioactive molecules and the advancement of novel techniques [4]. Microorganisms are an inexhaustible source of new bioactive molecules [5], with actinomycetes having produced nearly 10,000 antibiotics to date [6]. However, finding new compounds from traditional sources has become less likely in recent years [7], leading scientists to explore alternative microbial sources such as rare actinomycetes, insect-associated actinomycetes, and underrepresented taxa [8].

The genus *Gordonia* is a kind of rare actinomycetes [9]. Currently, most studies surrounding *Gordonia* bacteria focus on biodegradation [10,11]. There are few reports on their secondary metabolites, particularly those with antimicrobial properties. The first secondary metabolites to be identified from *Gordonia* were three novel steroid molecules called bendigoles A, B, and C [12]. Ana Patricia Gra et al. isolated the first three strains of *Gordonia* identified with antibacterial activity from *Erylus discophorus* sponges [13], and Park et al. found that *Gordonia* SP. KMC005 co-cultured with *Streptomyces tendae* KMC006 could produce a new antibacterial substance, gordonic acid [14]. However, no potential bioactive chemicals from *Gordonia* with anti-fungal action have been reported.

*P. americana* is a human health pest on a global scale and has been used in Chinese traditional medicine (TCM) for several thousand years [15]. *P. americana* extracts have been shown to improve immunity, blood circulation, and wound healing effects. Living in polluted areas has also made *P. americana* more resilient to microbial diseases [16], and bacteria in the *P. americana* gut are likely to produce antimicrobial compounds that aid the insect immune system.

In our previous studies, we found that *P. americana* gut-dwelling bacteria generate anti-bacterial chemicals that may be a source of new anti-bacterial compounds [[17], [18], [19], [20]]. The current investigation aimed to determine if secondary metabolites generated by *Gordonia*, isolated from the gut of *P. americana*, have any anti-fungal effects. The metabolites produced by *Gordonia* strain WA8-44 were purified, characterized, and assessed for their anti-fungal activities. Additionally, we used in silico models to assess their ADMET profiles and predict how they bind to known molecular targets in *C. albicans*.

## 2 Materials and methods

### 2.1 Sampling, pre-treatment, and isolation of actinobacteria

The *P. americanas* used in this study were collected from the campus of Guangdong Pharmaceutical University. Actinobacteria were isolated from the gut of *P. americana* using Lima et al.'s method [21]. Briefly, the *P. americana* samples were washed with sterile water, 75% ethanol, and 0.47 mol/L sodium hypochlorite. The gut was then ground and diluted to the required concentration (10, 5, 2.5 μg/mL). The samples were spread onto culture plates containing 0.25 mmol/L potassium dichromate and incubated at 28 °C for seven days. The visible colonies were isolated and purified based on their morphological characteristics.

### 2.2 Test pathogens for the current study

There were four fungi for testing; the fungi were *Candida albicans* ATCC 10231, *Trichophyton rubrum* ATCC 60836, *Aspergillus fumigatus* ATCC 96918, and *Aspergillus niger* ATCC 16404. All strains were obtained from Guangdong Microbial Culture Collection Center.

### 2.3 Screening of *gordonia* for anti-fungal activity

We evaluated the anti-fungal activity of *Gordonia* strains against *C. albicans* using the Oxford cup method. Briefly, *C. albicans* suspension (4 × 10^6^ CFU/mL) was swabbed onto PDA plates, and 200 μL of fermentation broth was added into an Oxford cup, followed by incubation for 24 h at 28 °C. We used amphotericin B (34.62 μmol/L) as a positive control and recorded the diameters of inhibition zones. For assessing the anti-fungal activity against *T. rubrum*, *A. fumigatus*, and *A. niger*, we performed plate confrontation assays [22]. Here, the *Gordonia* strain was inoculated on one side of PDA plates and incubated for four days at 28 °C. Subsequently, spores of *T. rubrum* ATCC 60836, *A. fumigatus* ATCC 96918, and *A. niger* ATCC 16404 were spotted on the other side of the plate, followed by incubation at 28 °C for 3–5 days. All experiments were conducted in triplicate.

### 2.4 Molecular identification and phylogenetic analysis of isolates

The micromorphological characteristics of the strains were observed using a scanning electron microscope (Quanta FEG 200, USA). Genomic DNA was extracted from WA8-44 using the GenElute Bacterial Genomic DNA Kit (Sigma). The 16 S rRNA gene was amplified using primers 27 F (5′-AGAGTTTGATCMTGGCTCAG-3′) and 1492 R (5′-TACGGCTACCTTGTTACGACTT-3′) from the 15 isolates, and the resulting PCR products were sequenced by Invitrogen Co. The obtained sequences were submitted to NCBI, and a phylogenetic tree was constructed using the neighbor-joining method with MEGA 5.0 software.

### 2.5 Bioassay-guided isolation and purification of anti-fungal compounds

An inoculum of the WA8-44 strain was cultured it in ISP-1 broth for three days at 28 °C 15 mL of the seed culture was then transferred to a 500 mL conical flask containing 300 mL ISP-2 medium and agitated on a rotary shaker at 28 °C for seven days. The fermentation broth was concentrated by centrifugation at 1708×*g* for 25 min, and the supernatant was extracted three times using ethyl acetate. The clear supernatant was then evaporated using a rotary flash evaporator and freeze-dried, yielding 12.0 g crude extract.

The crude extract was fixed on silica gel (300–400 mesh, 680 g) and fractionated through flash chromatography as ten fractions. Fractions 2 and 3 exhibited strong anti-fungal activity and were selected for further purification. Fraction 2 was fractionated using open-column chromatography with silica gel and yielded four subfractions (fractions 2–1 to 2–4). Fraction 2–3 was further purified using preparative RP-HPLC with a YMC-Pack ODS-A/S-5 μm/12 nm 250 × 10.0 mm column, a flow rate of 3 mL/min, MeOH/H_2_O (80% at 20 and 22 min), with UV detection at 210 nm. This led to the isolation of two compounds, A (182 mg) and B (20 mg). Fraction 3 was subjected to open-column chromatography using silica gel (gradient of PE/EA) and Sephadex LH-20 column chromatography with MeOH as the eluent. Compound C (29 mg) was then purified using preparative RP-HPLC with a YMC-Pack ODS-A/S-5 μm/12 nm 250 × 10.0 mm column, a flow rate of 3 mL/min, and MeOH/H_2_O (70% in 25 min), with UV detection at 210 nm.

### 2.6 Minimum inhibitory concentration testing

Following the Clinical and Laboratory Standards Institute (CLSI) guidelines, the M27-A3 method was used to determine the minimum inhibitory concentrations (MICs) of *C. albicans* [23], while the M38-A2 method was used for filamentous fungi [24]. To prepare the samples, *C. albicans* was diluted with RPMI1640 to a final concentration of 0.5–2.5 × 10^3^ spores/ml, *A. fumigatus*, and *A. niger* were diluted to 1 × 10^4^ spores/ml, and *T. rubrum* was diluted to 2 × 10^3^ spores/ml. The test samples were dissolved in DMSO and then diluted with RPMI1640 to a series of concentrations. Next, 100 μL of the test sample solutions were added to 100 μL of the cell suspension and incubated for 48 h. Each experiment was repeated three times.

### 2.7 Structural prediction of antifungal compounds

The NMR spectra of the chemicals were measured using a 600 MHz spectrometer (Brucker AVANCE III, 600 M, Germany), with tetramethyl silane (TMS) as the internal reference and dimethyl sulfoxide (DMSO) as the solvent. The compounds were injected separately into a Triple-quadrupole mass spectrometer (Thermo Scientific TSQ Endura™, USA) to obtain mass spectra. All solvents used for extraction were of LR grade, whereas HPLC-grade solvents were used for analysis.

Compound A, orange-red crystalline powder, ^1^H NMR (600 MHz, CDCl_3_) δ 7.60 (d, 1H); 7.35 (d, 1H); 5.20 (qd, 1H); 5.20 (qd, 1H); 4.60 (d, 1H); 4.51 (dd, 1H); 3.65 (d, 2H); 3.62 (d, 2H); 2.92 (s, 3H); 2.90 (s, 3H); 2.90 (s, 3H); 2.90 (s, 3H); 2.86 (s, 3H); 2.86 (s, 3H); 2.68 (s, 3H); 2.52 (s, 3H); 1.23 (d, 3H); 1.23 (d, 3H); 1.11 (d, 3H); 1.11 (d, 3H); 0.91 (d, 3H); 0.87 (d, 6H); 0.87 (d, 6H); 0.94 (d, 3H); 0.73 (d, 6H); 0.73 (d, 6H); ^13^C NMR (151 MHz, CDCl_3_) δ 179.10, 173.69, 173.39, 173.34, 173.32, 169.09, 168.63, 167.79, 167.70, 166.71, 166.63, 166.57, 166.42, 147.69, 145.93, 145.16, 140.56, 132.55, 130.43, 129.15, 127.85, 125.80, 113.62, 101.67, 75.13, 75.04, 71.42, 71.22, 58.93, 58.79, 56.62, 56.38, 55.23, 54.88, 51.46, 51.44, 47.67, 47.43, 39.36, 39.24, 35.03, 34.97, 31.85, 31.61, 31.33, 31.05, 29.36, 27.06, 26.99, 23.08, 22.90, 21.73, 21.64, 19.36, 19.32, 19.18, 19.15, 19.11, 19.07, 17.81, 17.38, 15.14, 7.84; HR-ESI-MS *m/z* 1277.43 [M+Na]^+^, HR-ESI-MS *m/z* 1255.49 [M+H]^+^.

Compound B, orange-red crystalline powder, ^1^H NMR (600 MHz, CDCl_3_) δ 8.24 (d, 1H); 7.71 (d, 1H); 7.66 (d, 1H); 7.14 (d, 1H); 6.55 (d, 1H); 5.93 (d, 1H); 5.25 (qb, 1H); 5.16 (qd, 1H); 4.72 (d, 1H); 4.57 (dd, 1H); 4.57 (d, 1H); 4.57 (d, 1H); 4.50 (dd, 1H); 3.98 (d, 1H); 3.87 (m, 1H); 3.83 (dd, 1H); 3.72 (m, 1H); 3.71 (dd, 1H); 3.64 (d, 1H); 3.64 (d, 1H); 3.58 (dd, 1H); 3.00 (s, 3H); 2.93 (s, 3H); 2.88 (s, 3H); 2.88 (s, 3H); 2.67 (m, 1H); 2.67 (m, 1H); 2.67 (m, 1H); 2.67 (m, 1H); 2.67 (m, 1H); 2.33 (d, 1H); 2.22 (m, 1H); 2.10 (m, 1H); 2.08 (m, 1H); 2.03 (m, 1H); 1.85 (dd, 1H); 1.26 (d, 3H); 1.14 (d, 3H); 1.12 (d, 3H); 1.12 (d, 3H); 0.95 (d, 3H); 0.95 (d, 3H); 0.90 (d, 3H); 0.85 (d, 3H); 0.74 (d, 3H); 0.74 (d, 3H). ^13^C NMR (151 MHz, CDCl_3_) δ 208.97, 179.17, 174.16, 173.67, 173.26, 172.83, 169.11, 168.87, 167.66, 167.63, 166.74, 166.46, 166.28, 166.07, 147.50, 146.09, 145.18, 140.67, 132.23, 130.51, 129.30, 128.07, 126.28, 113.79, 101.87, 74.90, 74.79, 71.58, 71.38, 58.69, 57.31, 56.60, 55.09, 54.89, 54.44, 53.01, 51.49, 51.45, 47.59, 42.05, 39.53, 39.35, 35.08, 34.93, 32.01, 31.85, 31.17, 27.17, 27.10, 23.12, 21.86, 21.75, 19.39, 19.36, 19.23, 19.20, 19.07, 18.97, 17.83, 17.29, 15.22, 7.93. HR-ESI-MS *m/z* 1291.17 [M+Na]^+^, HR-ESI-MS *m/z* 1269.41 [M+H]^+^.

Compound C, opalescent powder, ^1^H NMR (600 MHz, CDCl_3_) δ 10.63 (s, 1H), 9.09 (s, 1H), 8.66 (m, 1H), 8.52 (d, 1H), 8.03 (s, 1H), 7.85 (td, 1H), 7.34 (dd, 1H), 4.12 (s, 3H), 2.38 (s, 3H); ^13^C NMR (151 MHz, CDCl3) δ 167.37, 157.46, 155.15, 152.56, 148.93, 148.05, 137.18, 124.34, 122.17, 121.87, 103.71, 56.43, 18.52; HR-ESI-MS *m/z* 275.97 [M+H]^+^.

### 2.8 Scanning electron microscopy (SEM) analysis

The morphological changes induced in *C. albicans* by the active compounds were observed using SEM, as described by Jeyanthi et al. [25]. 4 × MIC of the active compounds were applied to *C. albicans* suspensions (1–5 × 10^5^ CFU/mL) for 48 h at 28 °C. The cells were then fixed in 2.5% glutaraldehyde at 4 °C overnight, washed three times with PBS for 20 min, and subsequently underwent a series of ethanol washes (30–100%). Finally, SEM images were obtained using a Quanta FEG 200 FESEM, which was operated at an accelerating voltage of 2–19 KV under standard operating conditions.

### 2.9 In-silico molecular docking studies

Nine molecular targets associated with antimicrobial biological activity in *C. albicans* were selected for the study. The crystal structures of dihydrofolate reductase (PDB ID: 1M78), secreted aspartic proteinase 5 (PDB ID: 2QZX) [26], 3-beta-glucanase (PDB ID: 4M80), lanosterol 14-alpha demethylase (PDB ID: 5V5Z) [27], chitin synthase 2 (PDB ID: 6LNK), fructose-1,6-bisphosphate aldolase (PDB ID: 7V6F) [28], and *N*-myristoyltransferase (PDB ID: 1IYL) [29], were obtained from the RCSB Protein Data Bank (https://www.rcsb.org/). Squalene epoxidase (AF ID: Q92206) [30] and thymidylate synthase (AF ID: P12461) were obtained from the AlphaFold Protein Structure Database (https://alphafold.ebi.ac.uk/). The 3D structures of the ligands, including Collismycin A (PubChem CID: 135482271), Actinomycin D (PubChem CID: 2019), and Actinomycin X_2_ (PubChem CID: 159855), were downloaded from the PubChem Database (https://pubchem.ncbi.nlm.nih.gov/). The 3D structures of the ligands were constructed and minimized using Chimera 1.16. The molecular docking was carried out using iGEMDOCK v2.1 with default parameter values [31], and protein-ligand interactions were analyzed using Biovia Discovery Studio Visualizer v2021.

### 2.10 In-silico drug-likeness and ADMET prediction of bioactive compounds

We utilized several web tools, including PreADMET (https://preadmet.qsarhub.com/) [32], pkCSM (https://biosig.lab.uq.edu.au/pkcsm) [33], ProTox-II (https://tox-new.charite.de/protox_II/) [34], and SwissADME (http://www.swissadme.ch/) [35], to predict drug-likeness and ADMET properties of the bioactive compounds.

## 3 Results

### 3.1 Isolation and screening

**Fig. 1:**
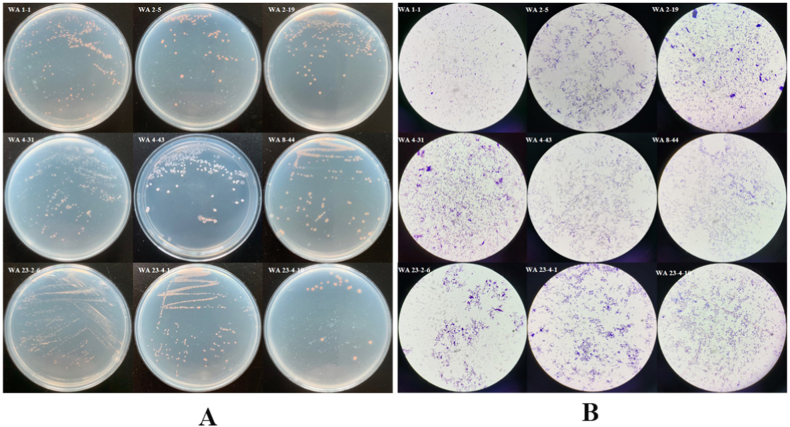
Morphological characteristics of strain *Gordonia*. **A** Results of identification of colonies of strain *Gordonia* on Gauze's medium. **B** Observation of culture plates by optical microscopy following Gram staining ( × 100).

15 strains of *Gordonia* were isolated from the gut of *P. americana* (as shown in Fig. 1AB). Among these strains, 8 strains demonstrated *anti*-*C. albicans* activity, 4 strains showed *anti*-*T. rubrum* activity, 3 strains displayed anti-*A. fumigatus* activity, and 3 strains exhibited anti-*A. niger* activity (as listed in Table 1). Notably, strain WA8-44 demonstrated significant activity against all the tested fungi. Therefore, strain WA8-44 was chosen for further investigation (as shown in Fig. 3ABCD and Table 1).

**Table 1:** Antimicrobial activity of *Gordonia* fermented liquid (mm growth inhibition, n = 3).

Strains	*C. albicans*	*T. rubrum*	*A. fumigatus*	*A. niger*
WA1-1	–	–	–	–
WA2-5	32.5 ± 3.6	+	–	–
WA2-19	18.7 ± 2.5	–	–	–
WA3-1-78	12.1 ± 1.9	–	–	–
WA4-31	30.8 ± 3.7	+	+	+
WA4-43	–	–	–	–
WA8-44	25.8 ± 3.2	+	+	+
WA12-1-1	16.6 ± 2.7	+	+	+
WA14-2-8	14.1 ± 1.5	–	–	–
WA20-4-4	–	–	–	–
WA23-2-5	12.2 ± 1.1	–	–	–
WA23-2-6	–	–	–	–
WA23-4-1	–	–	–	–
WA23-4-2	–	–	–	–
WA23-4-10	–	–	–	–
Amphotericin B	22.4 ± 2.8	+	+	+

Note: + Strain growth inhibited, - Strain growth is not inhibited.

### 3.2 Identification of potent isolate WA8-44

**Fig. 2:**
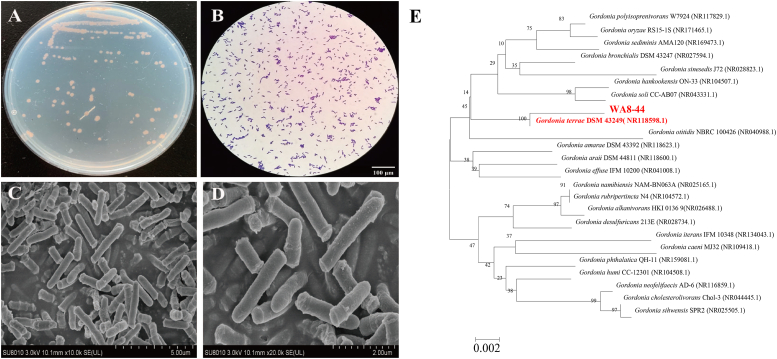
Morphological characteristics of *Gordonia* sp. WA8-44. **A** Colony morphology of *Gordonia* sp. WA8-44 on Gauze's No. 1 medium. **B** Gram stain of *Gordonia* sp. WA8-44. **C**&**D** Scanning electron micrograph of *Gordonia* sp. WA8-44. **E** Neighbor-Joining phylogenetic tree based on 16 S rRNA gene sequence showing the relationship between strain *Gordonia* sp. WA8-44 and representatives of some other related taxa.

*Gordonia* sp. WA8-44 exhibited good growth on Gauze's Agar No. 1 medium, with irregularly shaped and raised colonies that were either reddish-brown or milky yellow, with a moist and opaque surface (Fig. 2A). The strain was observed to be a gram-positive bacterium based on Gram staining results (Fig. 2B). Short, smooth, rod-shaped cell was observed using SEM (Fig. 2CD).

**Fig. 3:**
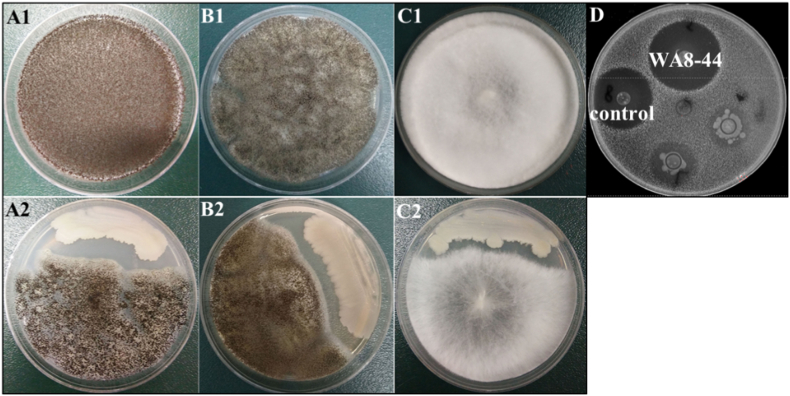
Anti-fungal activity of *Gordonia* sp. WA8-44. (A1) *A. niger*, (A2) Confrontation assay between *A. niger* and *Gordonia* sp. WA8-44; (B1) *A. fumigatus*, (B2) Confrontation assay between *A. fumigatus* and *Gordonia* sp. WA8-44; (C1) *R. rubrum*, (C2) Confrontation assay between *R. rubrum* and *Gordonia* sp. WA8-44; (D) Antifungal activity of *Gordonia* sp. WA8-44 against *C. albicans* using the Oxford cup method.

The 16 S rRNA sequence of strain WA8-44 was submitted to NCBI, and an accession number was obtained in GeneBank (MH605445). A phylogenetic tree of strain WA8-44, based on the Neighbor-Joining method, formed a single clade involving *Gordonia* terrae (NR_118,598) with a similarity of 99.55% (Fig. 2E). Molecular characterization confirmed that the strain belonged to *Gordonia terrae*.

### 3.3 Extraction, purification, and characterization

A bioassay-guided investigation was performed to isolate the active constituents from an ethyl acetate extract of strain WA8-44. Gradient elution was performed using different organic solvents and the activity of different fractions was determined using *C. albicans* ATCC 10231 as an indicator. The active components were further purified and the final purification of the sub-fractions was achieved by preparative RP-HPLC. In total, three active compounds were isolated and identified (Fig. 1S–9S). Compound A appeared as an orange-red crystalline powder and had a HR-ESI-MS *m*/*z* value of 1277.43 [M+Na]^+^ and 1255.49 [M+H]^+^. Compound B was also an orange-red crystalline powder and had a HR-ESI-MS *m*/*z* value of 1291.17 [M+Na]^+^ and 1269.41 [M+H]^+^. Compound C was an opalescent powder and had a HR-ESI-MS *m*/*z* value of 275.97 [M+H]^+^. The chemical structures of the purified compounds were elucidated based on HR-ESI-MS, ^1^H NMR, and ^13^C NMR. Consequently, Compounds A-C were identified as Actinomycin D, Actinomycin X_2_, and Collismycin A, respectively.

### 3.4 Anti-fungal activity of isolated bioactive compounds

**Fig. 4:**
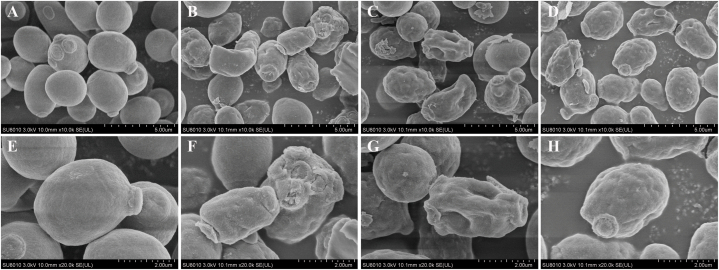
Scanning electron microscopy images of *C. albicans* treated and untreated with pure compounds. **A & E** Untreated *C. albicans*. **B & F** Actinomycin X_2_-treated *C. albicans*. **C& G** Actinomycin D-treated *C. albicans*. **D & H** Collismycin A-treated *C. albicans*.

The minimum inhibitory concentrations (MICs) of the isolated compounds are presented in Table 2. Actinomycin D, Actinomycin X_2_, and Collismycin A showed potent activity against *C. albicans* ATCC 10231, *T. rubrum* ATCC 60836, *A. fumigatus* ATCC 96918, and *A. niger* ATCC 16404, with MICs ranging from 3.19–101.96 μmol/L. This suggests that Actinomycin D, Actinomycin X_2_, and Collismycin A have the potential to be potent antifungal agents with broad spectra. The *anti*-*C. albicans* effect of Actinomycin D (Fig. 4CG), Actinomycin X_2_ (Fig. 4BF), and Collismycin A (Fig. 4DH) was observed through SEM analysis. The cell membrane structure of *C. albicans* ATCC 10231 was damaged after treatment with these compounds, which may have resulted in cell lysis.

**Table 2:** Antimicrobial activity of active compounds (MIC, μmol/L).

	Actinomycin D	Actinomycin X_2_	Collismycin A
*Candida albicans* ATCC 10231	3.19	25.21	7.26
*Aspergillus niger* ATCC 16404	101.96	100.84	58.11
*Aspergillus fumigatus* ATCC 96918	101.96	100.84	58.11
*Trichophyton rubrum* ATCC 60836	101.96	100.84	58.11

### 3.5 Molecular docking studies

In this study, molecular docking analysis was conducted to evaluate the potential binding affinity of Actinomycin D, Actinomycin X_2_, and Collismycin A to selected receptors. The receptors selected were *N*-myristoyltransferase, dihydrofolate reductase, Secreted aspartic proteinase 5, 1,3-Beta-Glucanase, lanosterol 14-alpha demethylase, chitin Synthase 2, fructose-1,6-bisphosphate aldolase, thymidylate synthase, and squalene epoxidase (Table 3). The results showed that Actinomycin D and Actinomycin X_2_ exhibited the highest affinity towards 1,3-Beta-glucanase with binding free energies ranging between −89.088 and −129.82 kcal/mol, and −78.579 and −118.283 kcal/mol, respectively. Collismycin A, on the other hand, showed the highest affinity towards Chitin Synthase 2 with a binding free energy of −94.578 kcal/mol, followed by squalene epoxidase (−86.692 kcal/mol), and 1,3-Beta-glucanase (−81.002 kcal/mol). These results suggest that Actinomycin D, Actinomycin X_2_, and Collismycin A have the potential to target these receptors and may be useful as antifungal agents.

**Table 3:** Molecular docking analysis between Actinomycin D, Actinomycin X_2_, and Collismycin A with receptors.

Receptors	ID	Docking predicted binding energy (Kcal/mol)		
		Actinomycin D	Actinomycin X_2_	Collismycin A
*N*-myristoyltransferase	1IYL	−97.636	−93.728	−79.694
Dihydrofolate reductase	1M78	−96.225	−93.095	−78.085
Secreted aspartic proteinase 5	2QZX	−89.088	−78.579	−59.835
1,3-Beta-Glucanase	4M80	−129.824	−118.283	−81.002
Lanosterol 14-alpha demethylase	5V5Z	−101.023	−86.989	−73.011
Chitin Synthase 2	6LNK	−127.663	−105.582	−94.578
Fructose-1,6-bisphosphate aldolase	7V6F	−99.196	−99.637	−66.599
Thymidylate synthase	P12461	−124.139	−106.425	−70.1723
Squalene epoxidase	Q92206	−99.508	−102.384	−86.692

**Fig. 5:**
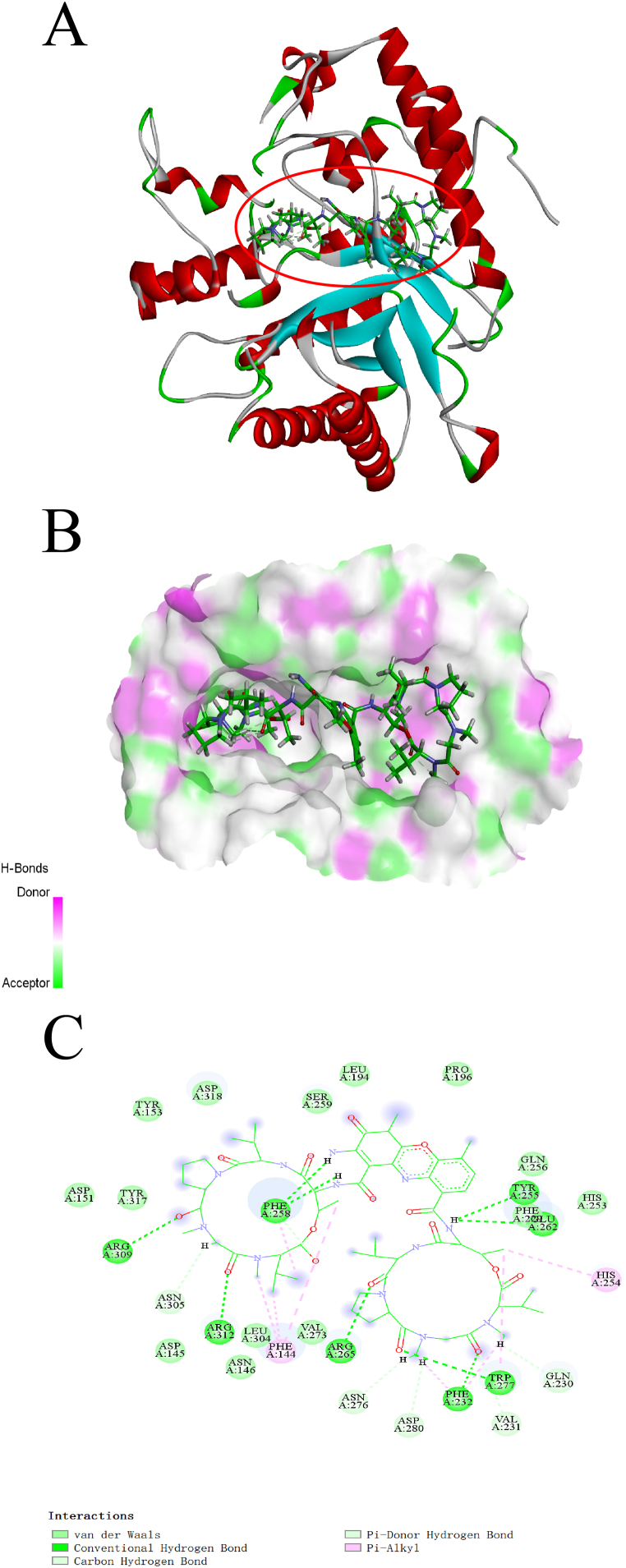
Binding complex and interaction visualization between 1,3-Beta-Glucanase (4M80) and Actinomycin D. **A** & **B** 3D interaction analyses of 1,3-Beta-Glucanase complexed with Actinomycin D. **C** 2D interactions analyses of 1,3-Beta-Glucanase complexed with Actinomycin D.

**Fig. 6:**
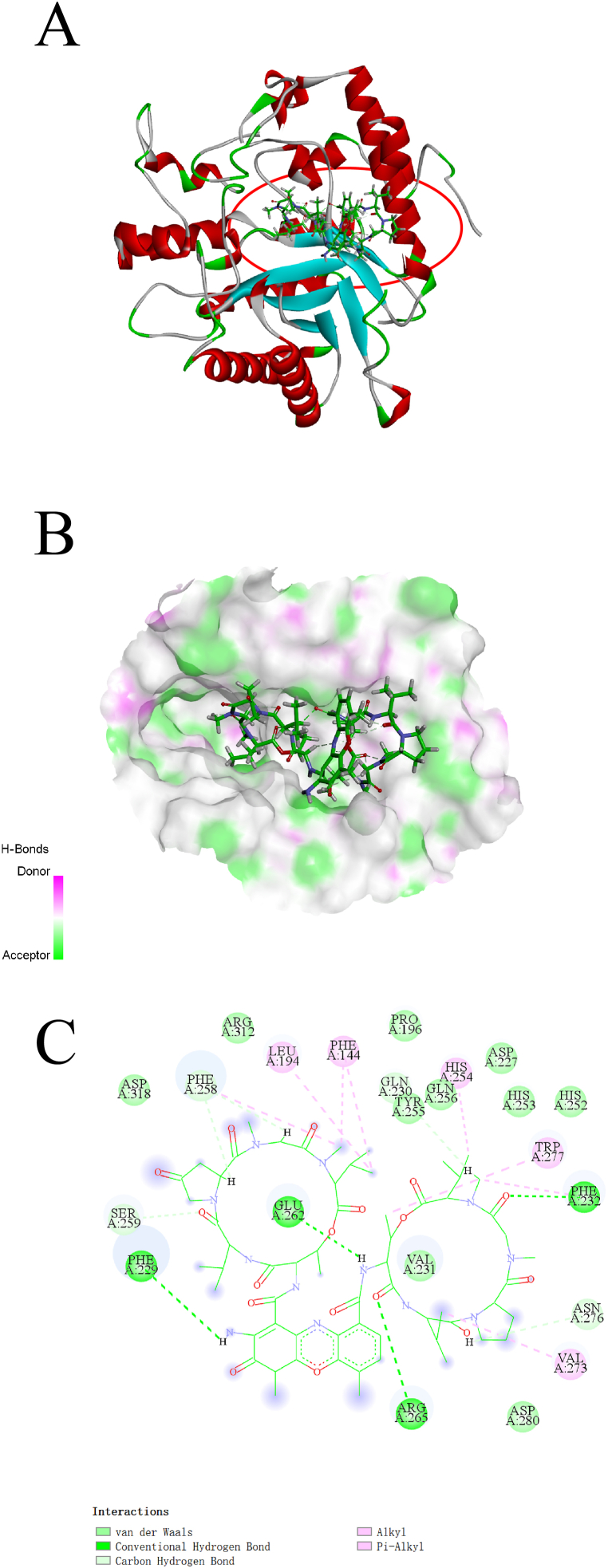
Binding complex and interaction visualization between 1,3-Beta-Glucanase (4M80) and Actinomycin X_2_. **A** & **B** 3D interaction analyses of 1,3-Beta-Glucanase complexed with Actinomycin X_2_. **C** 2D interactions analyses of 1,3-Beta-Glucanase complexed with Actinomycin X_2_.

**Fig. 7:**
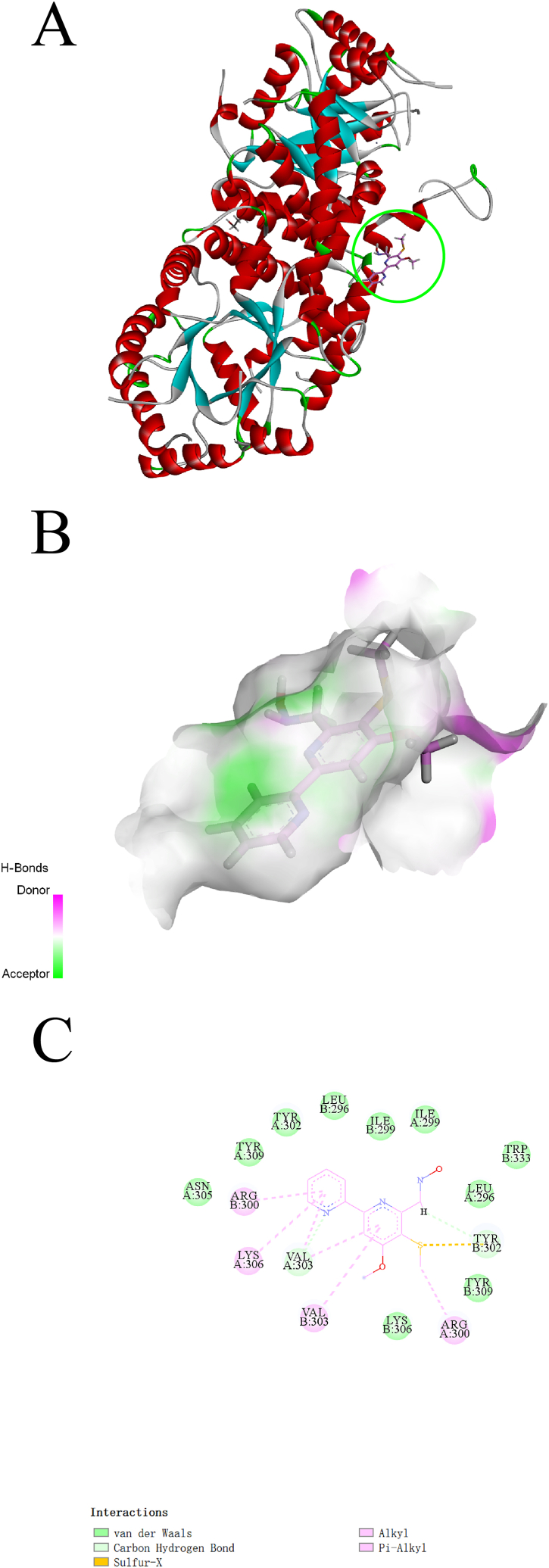
Binding complex and interaction visualization between Chitin Synthase 2 (6LNK) and Collismycin A. **A** & **B** 3D interaction analyses of Chitin Synthase 2 complexed with Collismycin A. **C** 2D interactions analyses of Chitin Synthase 2 complexed with Collismycin A.

We also provided visual representations of the interaction between these compounds and their respective receptors in Fig. 5, Fig. 6, Fig. 7AB, as well as detailed information about the intermolecular interactions in Table S1. The stability of the interactions was mainly due to the formation of H-bonds and hydrophobic interactions between the compounds and the receptor active sites. At 1,3-Beta-glucanase, Actinomycin D formed nine conventional H-bonds, five carbon H-bonds, and two hydrophobic interactions, while Actinomycin X_2_ formed four conventional H-bonds, four carbon H-bonds, and seven hydrophobic interactions (Fig. 5C). The researchers also noted that although Actinomycin D and Actinomycin X_2_ have a slight difference in chemical structure, the two compounds only have six same interactions (Fig. 6C). At Chitin Synthase 2, Collismycin A formed two carbon H-bonds with Tyr302 and Val303, and four hydrophobic interactions with Arg300, Arg300, Val303, and LysA306 (Fig. 7C). Overall, these findings suggest potential new targets for these compounds and provide insights for further drug design and development.

### 3.6 In-silico drug-likeness and ADMET prediction of bioactive compounds

In this study, Lipinski's rule of five was used to predict the drug-like properties of the bioactive compounds. According to Lipinski's rule of five, a molecule that is most likely to be developed as a candidate for an orally active medication should meet certain criteria, including a molecular weight less than 500 Da, no more than 10 hydrogen bonds, no more than 5 hydrogen H-Bond Donors, and MLogP<4.15. As shown in Table 4, the results indicate that Collismycin A complies with Lipinski's rule of five and can be strongly recommended as an oral drug.

**Table 4:** Lipinski's rule of five analysis.

Compounds	Molecular Formula	Lipinski's Parameters				
		Molecular Weight	MLogP	H-Bond Donor	H-Bond Acceptor	Violations
Actinomycin D	C_62_H_86_N_12_O_16_	1255.42	−2.99	5	17	2
Actinomycin X_2_	C_62_H_84_N_12_O_17_	1269.40	−3.74	5	18	2
Collismycin A	C_13_H_13_N_3_O_2_S	275.33	0.47	1	5	0

Pharmacokinetics refers to how a drug behaves after it is introduced into the body, with ADMET parameters being the factors influencing its pharmacokinetics. The ADMET parameters of the bioactive compounds in silico were presented in Table 5. Caco2 permeability of all compounds was above 0.9, which indicates that the compounds can be administered orally. The blood-brain barrier (BBB) plays a crucial role in maintaining homeostasis in the central nervous system (CNS) by preventing unrestricted movement of poisonous or harmful compounds, transit of nutrients, and removal of metabolites from the brain. However, both compounds cannot cross into the brain as their BBB permeability was above 0.3. Actinomycin D and Actinomycin X_2_ have a class II acute oral toxicity, which means they are fatal if swallowed, with LD_50_ values between 5 and 50 mg/kg, while Collismycin A has a class IV acute oral toxicity, which means it is harmful if swallowed, with LD_50_ values between 300 and 2000 mg/kg.

**Table 5:** In silico ADMET properties of each compound.

Parameters	Actinomycin D	Actinomycin X_2_	Collismycin A
Absorption (A)			
Caco2 permeability	1.464	1.486	1.21
Distribution (D)			
BBB permeability (<-1)	−1.383	−1.408	−0.66
Metabolism (M)			
CYP1A2 inhibitor	No	No	No
CYP2C19 inhibitor	No	No	No
CYP2C9 inhibitor	No	No	No
CYP2D6 inhibitor	No	No	No
CYP3A4 inhibitor	No	No	No
Excretion (E)			
Clearance	−1.374	−1.448	0.757
Renal OCT2 substrate	No	No	No
Toxicity (T)			
Cytotoxicity	Yes	Yes	No
AMES toxicity	No	No	No
Hepatotoxicity	Yes	Yes	Yes
Skin Sensitisation	No	No	No
Oral Toxicity (LD50) (mg/kg)	7	7	1000
Acute oral toxicity	Class II	Class II	Class IV

## 4 Discussion

Rare actinomycetes have recently gained popularity in research, and several valuable compounds have been discovered from them [36]. Examples include vancomycin from *Amycolatopsis* [37], macrolides from *Micromonospora* [38], and erythromycin from *Saccharopolyspora* [39]. *Gordonia* is a member of the rare actinomycetes family that can be found in various environments such as the human body, soil, sewage, and oil wells [40]. However, it is rarely found in insects. In this study, 15 strains of *Gordonia* were isolated from the gut of *P. americana* for the first time, which provides new sources of *Gordonia*.

Insects are the largest group in nature and they harbor a large number of symbiotic bacteria in their bodies. It has been suggested by many researchers that gut-inhabiting bacteria protect their insect hosts against pathogens by synthesizing specific antimicrobial compounds [[41], [42], [43]]. For instance, Nurdjannah et al. found that *Bacillus cereus* from *Apis dorsata* gut produced surfactin, fengycin, and iturin A that exhibited potent *anti*-*Neisseria gonorrhoeae* activity [44]. *Pleosporales* sp. BYCDW4 from *Odontotermes formosanus* produced 5-hydroxyramulosin and biatriosporin M, which showed strong antibacterial effects against *E. coli*, *C. albicans*, *B. subtilis*, and *S. aureus* [45]. It is widely acknowledged that microorganisms isolated from insects are crucial sources for discovering and producing bioactive compounds.

The microbiomes of insects hold great promise for developing new antibiotics to treat fungal infections. In a study by MG Chevrette et al., insect-associated actinomycetes showed significantly greater activity against fungi and a wider range of biosynthetic capabilities compared to soil-associated actinomycetes, as determined through ecologically optimized bioassays, genomic, and metabolomic analyses [46]. While previous studies have not reported the anti-fungal activity of *Gordonia* isolated from different environments, our research found that 53.3% of *Gordonia* strains isolated from the gut of *P. americana* exhibited anti-fungal effects. This discovery highlights a new source of microorganisms for exploring novel natural anti-fungal products.

Out of all the isolates, WA8-44 showed robust anti-fungal activity against common human pathogens. Therefore, WA8-44 was selected for further investigation. Through morphological characteristics, molecular identification, and 16 S rRNA phylogenetic tree analysis, WA8-44 was identified as *Gordonia terrae* (GenBank Accession Number: MH605445). Using bioassay-guided isolation methods, three active anti-fungal compounds were separated from the fermentation broth of WA8-44. The purified compounds were chemically characterized through MS, ^1^H NMR, and ^13^C NMR. The three active anti-fungal compounds were identified as Collismycin A, Actinomycin D, and Actinomycin X_2_.

Collismycin A is a natural product that is known to have cytotoxic activity against cancer cells and is typically produced only by *Streptomyces* [47]. However, our study discovered that *Gordonia* also produces Collismycin A, which showed remarkable activity against fungi. While previous studies have not reported the anti-filamentous fungi activity of Collismycin A, our findings reveal its potential as an effective anti-filamentous fungi agent. Additionally, Actinomycin D and Actinomycin X_2_, two chromopeptide lactone antibiotics with various biological activities, including anti-cancer [48], anti-bacterial, anti-fungal [49], and anti-leishmanial properties [50], were also identified in *Gordonia*. These compounds are typically produced by *Streptomyces* [51] and, in some cases, *Micromonospora* [52], but this study demonstrated that they can also be produced by *Gordonia*. The MIC assay showed that all three active compounds had significant anti-fungal activity against *C. albicans*, *T. rubrum*, *A. fumigatus*, and *A. niger*, with MICs ranging from 3.19–101.96 μmol/L.

Molecular docking is a powerful computational method used to predict the binding affinities and conformations of drug candidates [53,54]. In this study, in-silico virtual screening was employed to determine the mechanism of action of three antifungal bioactive compounds derived from *Gordonia* sp. WA8-44 against *C. albicans*. Nine modeled protein targets from *C. albicans* were docked with the bioactive compounds, and the results showed that Actinomycin D and Actinomycin X_2_ were the most potent against 1,3Beta-Glucanase. This protein is a potential therapeutic target for treating Candida infections because it is responsible for most of the glucanase activity in the Candida cell wall [55]. Although Actinomycin X_2_ and Actinomycin D are similar in chemical structure, Actinomycin D exhibited stronger *anti*-*C. albicans* activity due to its six additional H-bonding interactions with 1,3Beta-Glucanase [56]. Likewise, in the Hamdoon's study, methylated taxifolin demonstrated enhanced binding affinity towards various pro- and antiapoptotic proteins compared to taxifolin, thereby displaying heightened anticancer efficacy [57]. Although previous studies have reported the anti-*Candida albicans* effect of Collismycin A, its mechanism of action is still unclear [58]. In *Candida albicans*, Chitin Synthase II is primarily responsible for synthesizing the primary septum and is considered a potential target for antifungal drugs [59]. These results provide insights into the possible specific mechanisms of action of these compounds and could guide future experimental investigations.

Drug development is a time-consuming and costly process, and inadequate pharmacokinetics is a significant cause of failure in clinical trials [60]. Therefore, ADME data, which provide information on a drug's absorption, distribution, metabolism, and excretion, are becoming increasingly crucial in drug development. In-silico techniques offer a cost-effective way to filter and optimize drug candidates in the early stages of drug development since experimental acquisition of ADME data can be expensive. In this study, an in-silico assessment of the ADME properties of the three bioactive compounds was conducted. The Caco2 permeability values of all compounds were greater than 0.9, suggesting their suitability for oral administration. The BBB permeability of all three substances was low, indicating that none of them would have any significant negative impact on the central nervous system. In the metabolic process of drugs, CYP enzymes, including CYP450 inhibitors, play a central role. None of the three drugs were predicted to inhibit P450, indicating that they would not present drug-drug interaction problems with medications targeting those CYP enzymes. Consistent with previous reports in the literature, Actinomycin D and Actinomycin X_2_ were predicted to have moderate toxicity [48]. The results obtained from the in-silico ADMET studies predicts Collismycin A as a potent oral drug candidate due to its low toxicity and compliance with the Lipinski rule of five.

This study represents the first attempt to isolate anti-fungal secondary metabolites from *Gordonia* associated with *P. americana*. A total of 15 strains of *Gordonia* were isolated from the gut of *P. americana*, and 53.3% of them exhibited anti-fungal activity. Among these strains, three anti-fungal compounds (Collismycin A, actinomycin D, and actinomycin X_2_) were identified and purified from the secondary metabolites of *Gordonia* sp. WA8-44. Notably, Collismycin A was isolated from *Gordonia* for the first time, and its anti-filamentous fungi activity was firstly identified in this study. Based on the predicted ADMET properties, Collismycin A demonstrated good oral bioavailability and low toxicity, making it a promising candidate for the development of orally active drugs.

## Author contribution statement

Wenbin liu, Ph.D.; Xiaobao Jin: Conceived and designed the experiments; Contributed reagents, materials, analysis tools or data; Wrote the paper.

Ertong Li: Analyzed and interpreted the data.

Lingyan Liu; Fangyuan Tian; Xiongming luo; Yanqu Cai: Performed the experiments.

Jie Wang: Conceived and designed the experiments.

## Data availability statement

Data included in article/supp. Material/referenced in article.

## Additional information

Supplementary content related to this article has been published online at [URL].

## Funding statement

This work was funded by the Guangzhou Science and technology planning project (No. 202201010357), the 10.13039/501100003453Guangdong Natural Science Foundation (No. 2020A1515011097), the Public Welfare Research and Capacity Building Project of Guangdong Province (2017A020211008), and the Key Projects of Basic Research and Applied Basic Research of Guangdong Province Normal University (No. 2018KZDXM041).

## Declaration of competing interest

The authors declare that they have no known competing financial interests or personal relationships that could have appeared to influence the work reported in this paper.
