# Supplementary material for: Antifungal activity of compounds from Gordonia sp. WA8-44 isolated from the gut of Periplaneta americana and molecular docking studies
Source: Heliyon. 2023 Jul 4;9(7):e17777. doi: 10.1016/j.heliyon.2023.e17777 (PMC10395128; doi:10.1016/j.heliyon.2023.e17777)
Supplement: Multimedia component 1 [file mmc1.docx]

# Antifungal activity of compounds from *Gordonia* sp. WA8-44 isolated from the gut of *Periplaneta americana* and molecular docking Studies

Wenbin Liu^1^· Ertong Li^1^· Lingyan Liu^2^· Fangyuan Tian^1^· Xiongming Luo^1^·Yanqu Cai^1^·Jie Wang^1^· Xiaobao Jin^1^

**Table S1** Analysis of the compounds’ interactions with receptors

| Targets | Actinomycin D | | Actinomycin X_2_ | | Collismycin A | |
| --- | --- | --- | --- | --- | --- | --- |
|  | Residues involved in H-bonding | Residues involved in  hydrophobic interactions | Residues involved in H-bonding | Residues involved in  hydrophobic interactions | Residues involved in H-bonding | Residues involved in  hydrophobic interactions |
| N-myristoyltransferase | Tyr:A119; Tyr:A335; Leu:A337; Phe:A339; Gly:A351; Ile:A352; | Leu:A235; Leu:A350; Ala:A353; Val:A390; | Gly:A35;Tyr:A119; Tyr:A335; Leu:A337; Phe:A339; Ile:A352; | Leu:A235; Leu:A350; Ala:A353; Val:A390; | Gln: A226; | Tyr:A225; Tyr:A354; |
| Dihydrofolate reductase | Gly:A23; Lys:A57; Thr:A147; | Lys:A57; Arg:A79; Ala:A115; | Thr:A58; Glu:A60; | Ile:A19; Lys:A24; Lys:A57; Ala:A115; | Thr:A58; Gly:A114; Ala:A115; Thr:A147; | Lys:A57; Ile:A117; |
| Secreted aspartic proteinase 5 | Thr:A33; Gly:A34; Ser:A35; Ser:A36; Trp:A39; Lys:A83; Lys:A193; Ser:C206; Leu:A216; | Ile:A82; Lys:A193; Ala:C205; | Asp:A86; Tyr:A225 | Tyr:A89; Val:C203; Ile:A305; Tyr:A225; | Leu:A217; | Lys:A193; Leu:A216; |
| 1,3-Beta-Glucanase | Gln:A230; Val: A231; Phe:A232; Tyr: A255; Phe: A258(2); Glu: A262; Arg:A265; Glu: A262; Trp: A277; Asp:A280; Phe: A299; Asn:A305; Arg:A309; Arg:A312; | Phe:A144; His: A254; | Phe:A229;Phe:A232;Tyr:A255; Phe:A258; Ser:A259; Arg:A265; Asn:A276; | Phe:A144; Leu:A194; Phe:A232; His:A254; Phe:A258; Val:A273; Trp:A277; | Asp:A150; Phe:A258; | Arg: A150; |
| Lanosterol 14-alpha demethylase | Val:A125; Phe:A228; Ile:A231; | Val:A125; Ile:A231; Ile:A302; Met:A306; His:A310; | Phe:A126; Asn:a232; | Lys:A119; Ile:A131; Ile:A302; |  | Leu:A87; Leu:A88; Pro:A230; |
| Chitin Synthase 2 | Asn:B52; Gly:B72; Arg:A335; Lys:A339; | Arg:A50; Lys:B71; Lys:A343; Ala:A346; | Lys:B8; Lys:B15; Asp:B16; Thr:B134; | Pro:B3; Lys:B15; | Tyr:B302; Val:A303; | Arg:A300; Arg:B300; Val:B303; Lys:A306; |
| Fructose-1,6-bisphosphate aldolase | His: A225; | His:A109; Val:A224; | His: A225; | His:A109; Val:A224; | His: A109 | Gly: A265; |
| Thymidylate synthase | Phe: A61; | Lys:A59; Phe:A61; Lys:A63; Ile:A89; Phe:A223; Pro:A308; Met:A311; Met:A313; | Arg: A29; | Arg:A29; Phe:A168; Tyr:A256; | Gly:A220; Asn:A224; | Ile:A89; Leu:A219; Met:A313; |
| Squalene epoxidase | Ser:A120; Thr:A121; | Pro:A121; | Asn:A492; Arg:A496; | Tyr:A313; Pro:A488; Tyr:A489; | Val:A34; Arg:A43; | Arg:A36; Val:A164; Ile:A202; Tyr:A203; |


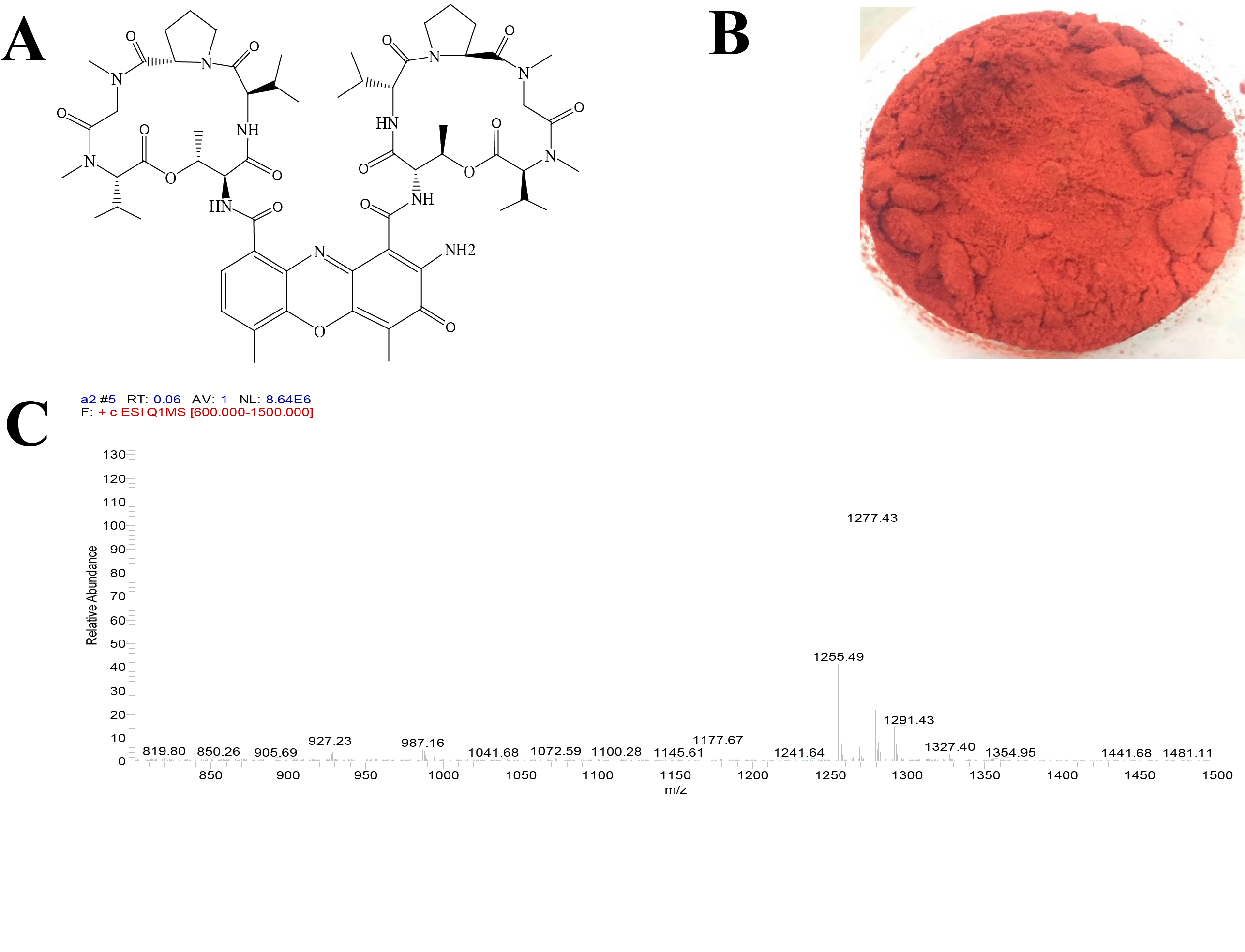


**Fig. 1S** Structure identification of the purified compound A. (A) Molecular structure of the compound. (B) Appearance of the compound A. (C) Mass spectrometry.


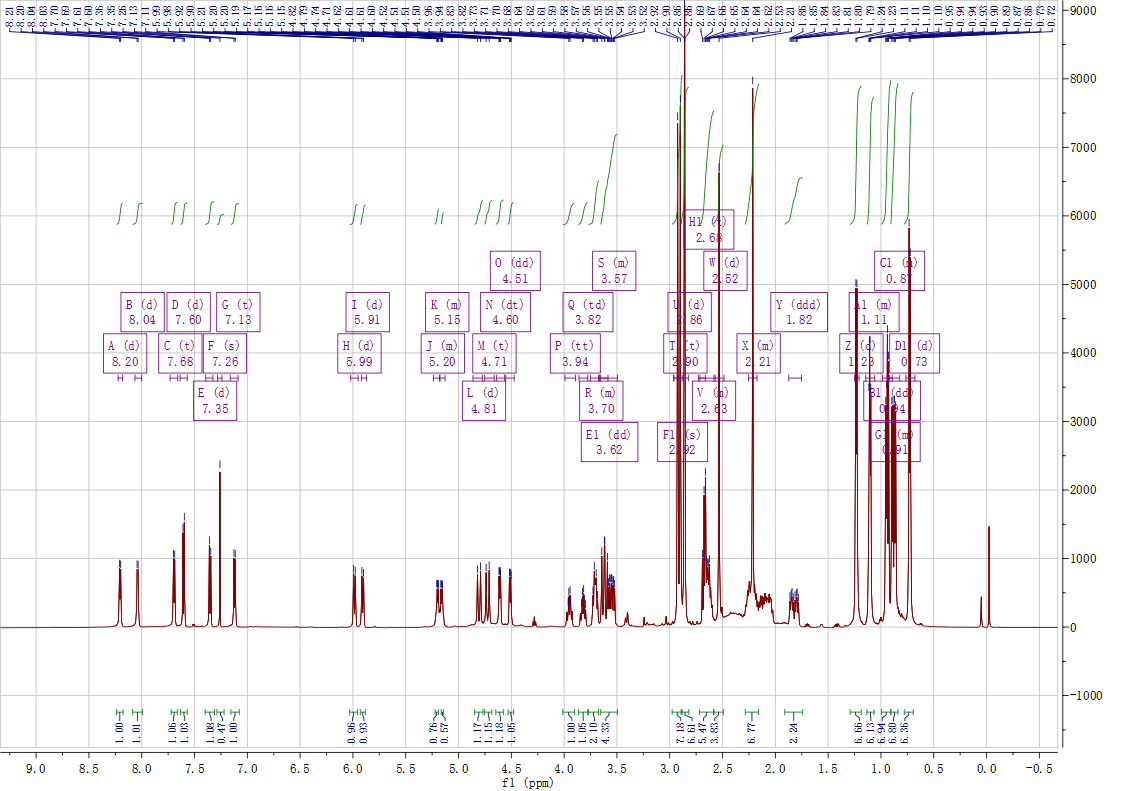


**Fig. 2S** Proton (^1^H) nuclear magnetic resonance of the compound A.


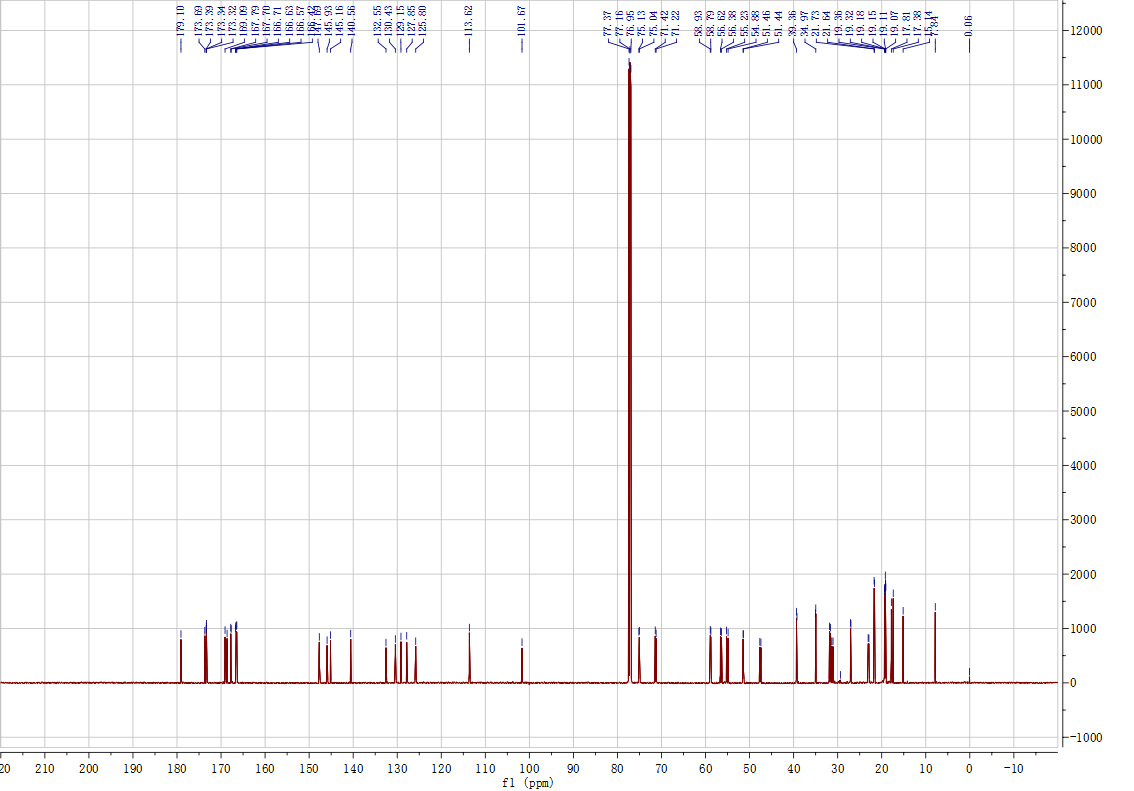


**Fig. 3S** Carbon (^13^C) nuclear magnetic resonance spectrum of the compound A in CDCl_3_.


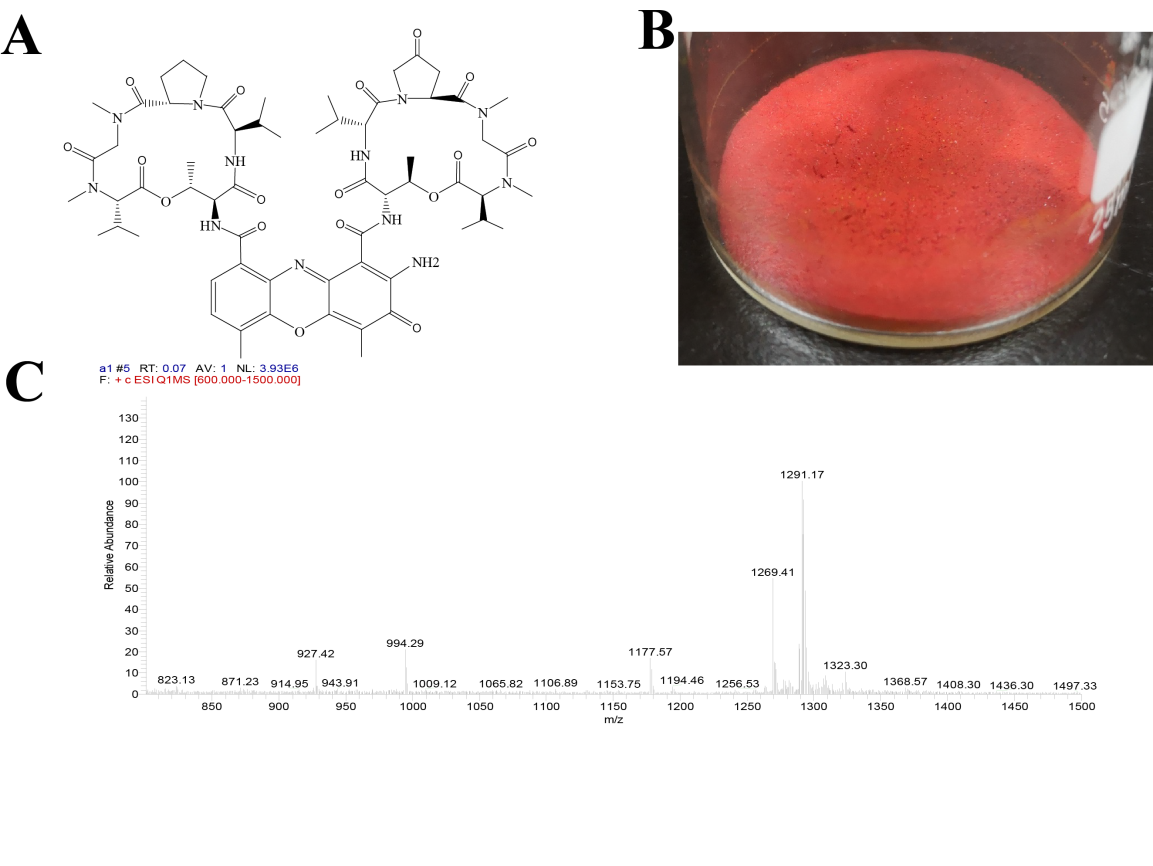


**Fig. 4S** Structure identification of the purified compound B. (A) Molecular structure of the compound. (B) Appearance of the compound B (C) Mass spectrometry.


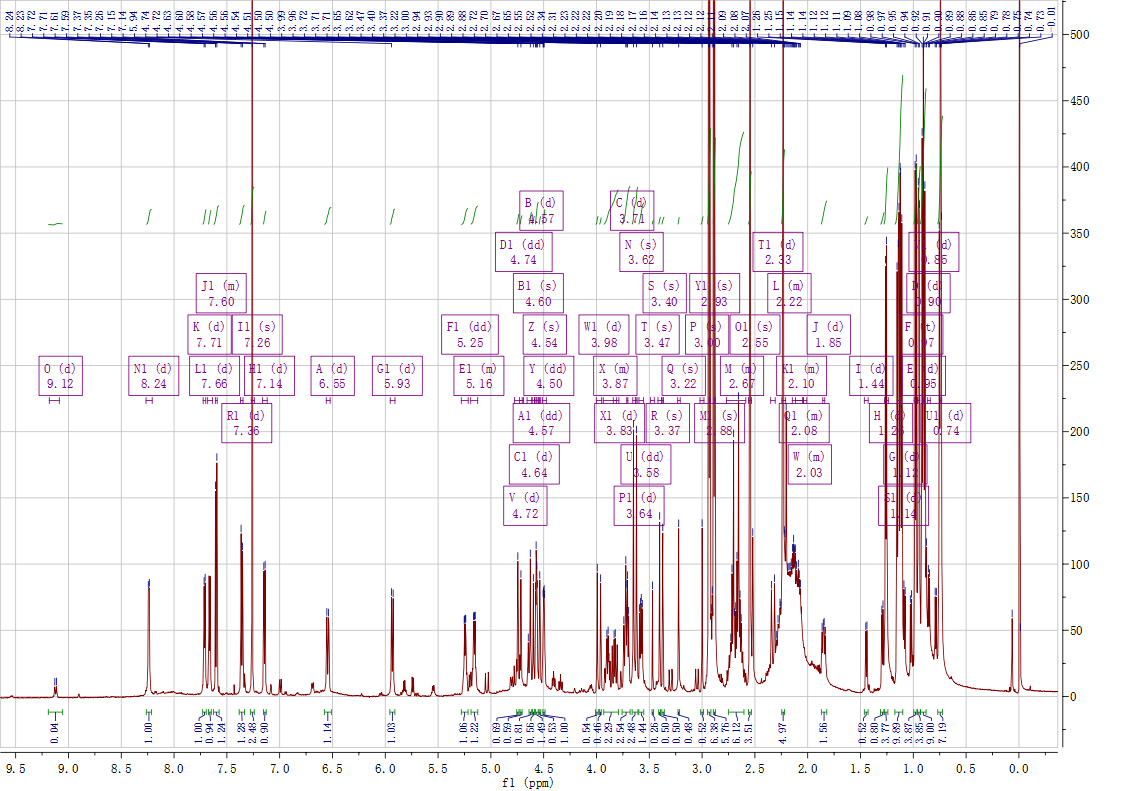


**Fig. 5S** Proton (^1^H) nuclear magnetic resonance of the compound B.


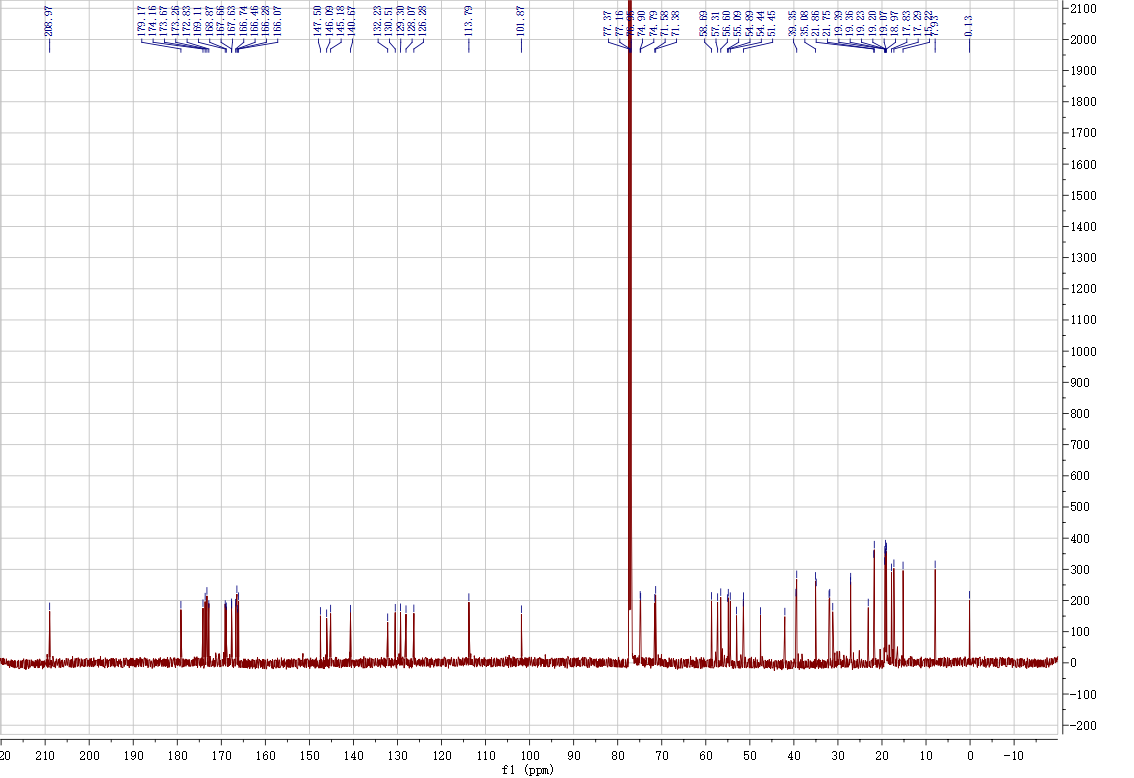


**Fig. 6S** Carbon (^13^C) nuclear magnetic resonance spectrum of the compound B in CDCl_3_.


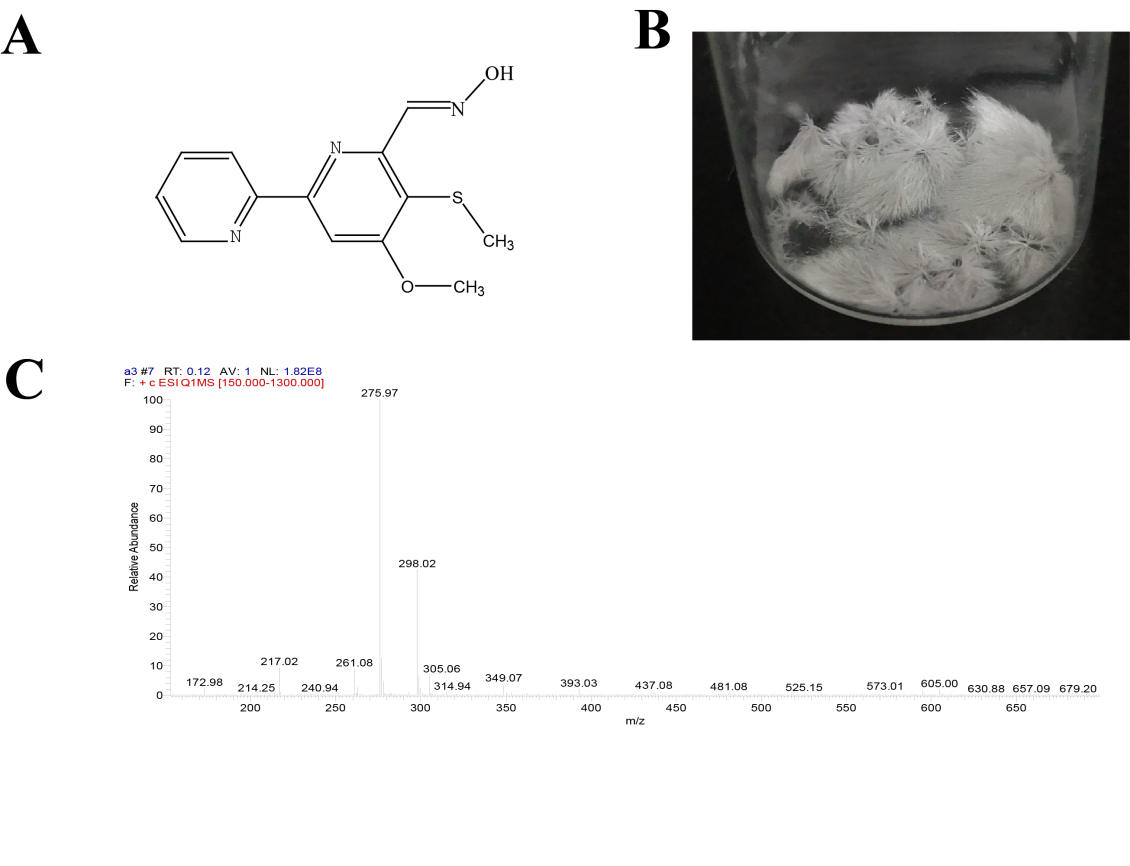


**Fig. 7S** Structure identification of the purified compound C. (A) Molecular structure of the compound. (B) Appearance of the compound C. (C) Mass spectrometry.

**
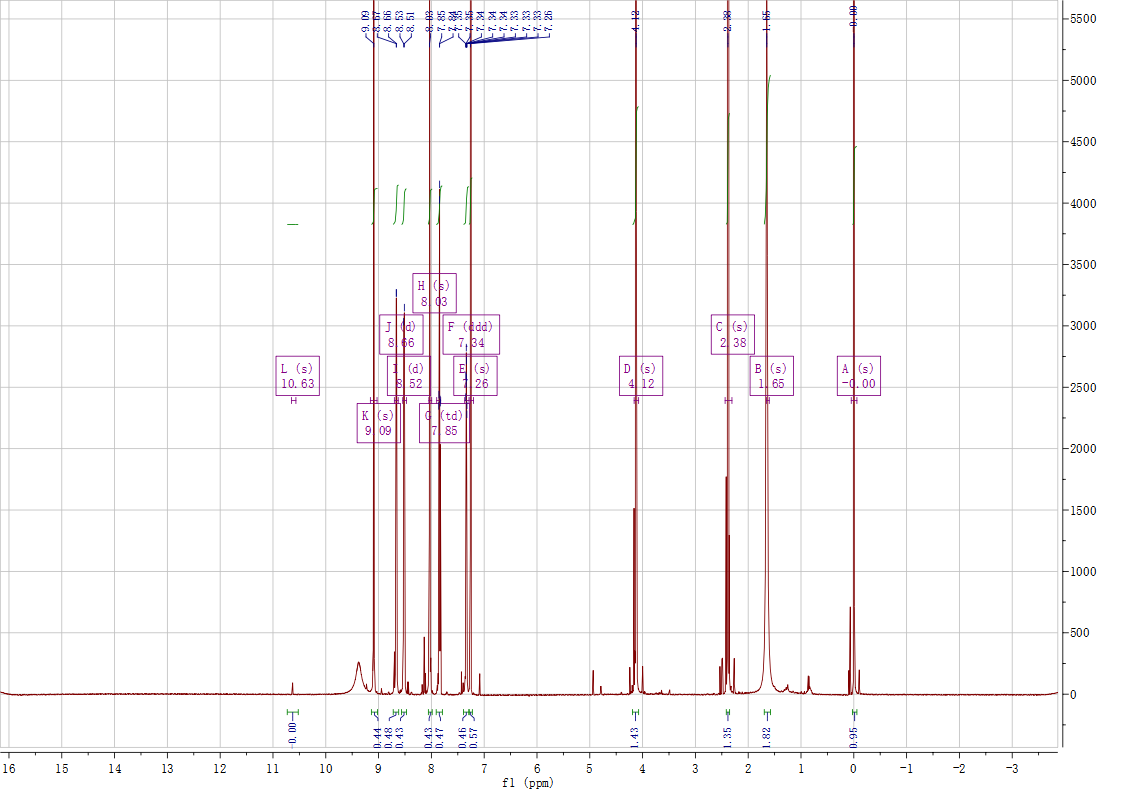
Fig. 8S** Proton (^1^H) nuclear magnetic resonance of the compound C.

**
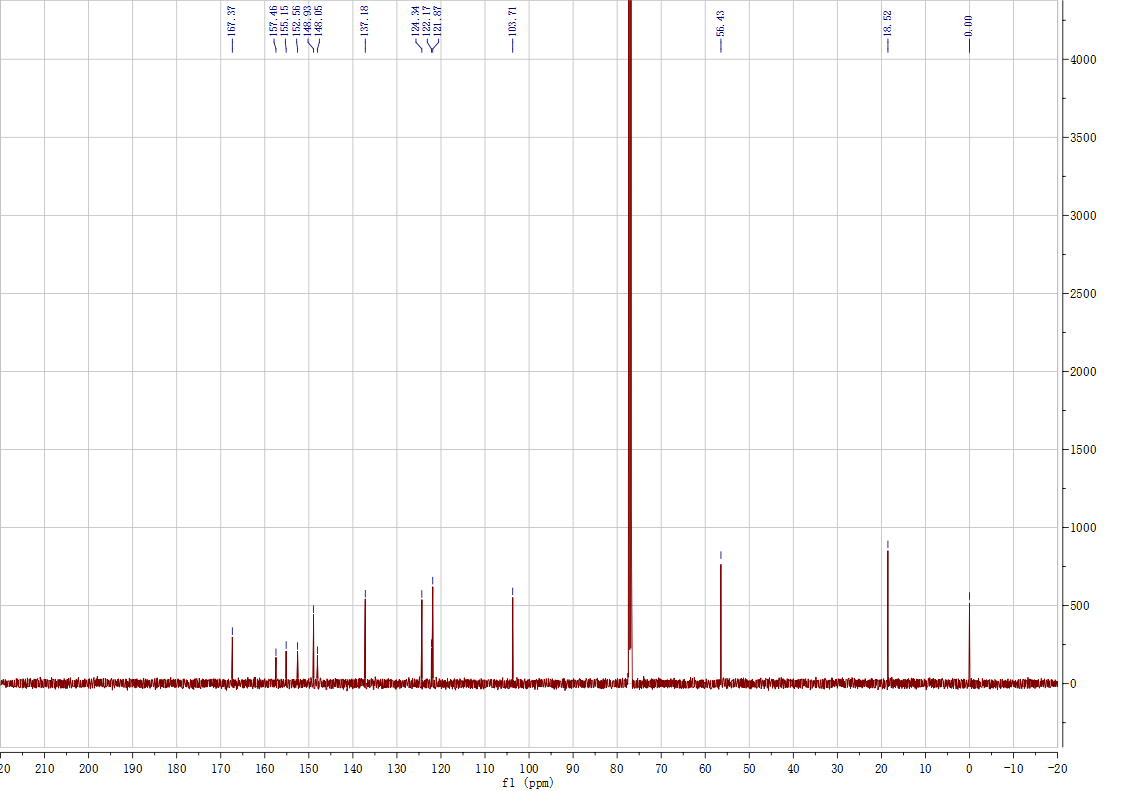
**

**Fig. 9S** Carbon (^13^C) nuclear magnetic resonance spectrum of the compound C in CDCl_3_.
